# Effects of Nitrogen Supply on Water Stress and Recovery Mechanisms in Kentucky Bluegrass Plants

**DOI:** 10.3389/fpls.2017.00983

**Published:** 2017-06-08

**Authors:** Shah Saud, Shah Fahad, Chen Yajun, Muhammad Z. Ihsan, Hafiz M. Hammad, Wajid Nasim, Amanullah, Muhammad Arif, Hesham Alharby

**Affiliations:** ^1^College of Horticulture, Northeast Agricultural UniversityHarbin, China; ^2^College of Plant Science and Technology, Huazhong Agricultural UniversityWuhan, China; ^3^Cholistan Institute of Desert Studies, The Islamia University of BahawalpurBahawalpur, Pakistan; ^4^Department of Agronomy, The Islamia University of BahawalpurBahawalpur, Pakistan; ^5^Department of Environmental Sciences, COMSATS Institute of Information TechnologyVehari, Pakistan; ^6^Department of Agronomy, Faculty of Crop Production Sciences, The University of AgriculturePeshawar, Pakistan; ^7^Department of Biological Sciences, Faculty of Science, King Abdulaziz UniversityJeddah, Saudi Arabia

**Keywords:** Kentucky bluegrass, drought, nutritional soil, transmission electron microscopy, C:N ratio, homogenous and heterogeneous plots

## Abstract

Non-irrigated crops in temperate and irrigated crops in arid regions are exposed to an incessant series of drought stress and re-watering. Hence, quick and efficient recuperation from drought stress may be amongst the key determinants of plant drought adjustment. Efficient nitrogen (N) nutrition has the capability to assuage water stress in crops by sustaining metabolic activities even at reduced tissue water potential. This study was designed to understand the potential of proper nutrition management by studying the morphological and physiological attributes, and assimilation of nitrogen in Kentucky bluegrass under drought stress. In present study, one heterogeneous habitat and four treatments homogenous habitats each with four replications were examined during field trial. Drought stress resulted in a significant reduction in the nitrogen content of both mother and first ramets, maximum radius, above and below ground mass, number of ramets per plot, leaf water contents and water potential and increased the carbon content and the C:N ratio in both homogenous and heterogeneous plots compared to well-watered and nutritional conditions. Observation using electron microscopy showed that drought stress shrunk the vessel diameter, circumference and xylem area, but increased the sieve diameter, and phloem area in the leaf crosscutting structure of Kentucky bluegrass, first, second, and third ramet leaf. Thus, it can be concluded that water stress markedly reduced all the important traits of Kentucky bluegrass, however, proper nutritional management treatment resulted in the best compensatory performance under drought assuaging its adversity up to some extent and may be considered in formulating good feasible and cost-effective practices for the environmental circumstances related to those of this study.

## Introduction

By the end of this century, global surface average temperature will rise about 1.1–6.4°C according to the fourth assessment report published by IPCC (Fischlin et al., 2007). This means that a warming above 3°C would eradicate thoroughly fixed carbon function of worldwide terrestrial vegetation, shift a net carbon source. It is estimated that drought stress would be intensified with this global warming due to enhancement in evapotranspiration rates, and on other hand will also escalate the water stress frequency and intensity with a rise from 1 to 30% in acute drought land area by 2100 (Fischlin et al., 2007); which might disturb the positive influences from the raised CO_2_ concentration, further restraining the structure and function of the terrestrial ecosystem. Precipitation regimes with its distribution and amount may estimate by the global climate models, nevertheless the complex reactions of terrestrial ecosystem to climate variation may severely influence the forecast precision. In agriculture, drought stress can be described by the shortage of soil water in an environment as a result of rainfall being rarer than normal (UNISDR, 2009). It is deemed to be one of the major reasons for diminishing plant growth attributes among different abiotic aspects in several regions of the world and is gradually rising due to global warming issues (Yang et al., 2010). Globally, about one-third of the land area is subjected to drought dilemmas, and only in China is this ratio more than 47% (Wei et al., 1997) because future predictions for the continuous changing global environmental circumstances predict increasing severity and incidence of drought in the coming decades (Dai, 2013). Engineering and breeding more effectual and well adapted drought crop cultivars is becoming increasingly significant. Numerous alterations in morphological, metabolic, or/and physiological traits are induced by drought stress in plants. At the preliminary stage of plant growth and development, it adversely influences both plant elongation and growth expansion (Kusaka et al., 2005; Shao et al., 2008). In several species, diminishing leaf growth and leaf area and a greater root/shoot ratio has been documented in response to drought (Jaleel et al., 2009). Severe drought stress poses injurious outcomes on plant water relations, photosynthesis, ion uptake, nutrient metabolism and the partitioning of assimilates (Farooq et al., 2009; Jaleel et al., 2009; Saud et al., 2013, 2014, 2016). Stomatal closure and turgor losses that occur under drought stress are due to a sporadic water supply from the xylem to the surrounding elongating cells (Taiz and Zeiger, 2006). Therefore, both stomatal and non-stomatal restraints are deemed to be the core causes of decreased photosynthesis activity under drought stress (Farooq et al., 2009).

Under drought stress, plant reactions are extremely intricate and fluctuate amongst plant species and growth phases, along with water limitation durations (Farooq et al., 2009; Fahad and Bano, 2012; Aslam et al., 2014; Fahad et al., 2015). Nevertheless, root, shoot growth attributes along with the leaf area are significantly hampered by drought stress with an ensuing decline in the growth and development of plants (Anjum et al., 2011). Drought stress severely influenced plant water status by reducing the water potential and the relative water content (RWC) in wheat (Siddique et al., 2001). In response to drought stress, plants develop many adaptive strategies including escape, avoidance and tolerance mechanisms (Chaves et al., 2003). In fact, these strategies in plants under drought conditions may play a combined role, not absolute separate (Fry and Huang, 2004). Morphological plasticity, water physiological integration or gene regulation of plants could be possible to response to drought during the same acclimation period (Jackson et al., 2000). Optimal nitrogen application also plays a critical role in combating drought (Marschner, 1995). Plant roots have to absorb more water to be able to take up the same amount of nitrogen for metabolism from soil under low nitrogen concentrations. Vice versa, in conditions of drought stress, plant roots are unable to get optimal amounts of nitrogen from soil, which has negative effects on plants growth by disturbance physiological metabolisms (Waraich et al., 2011).

Plants match their rate of growth according to resource accessibility [for example, water, light, and nitrogen (N)] using a number of acclimation mechanisms. Recognizing the principal strategies and growth attributes that describe how plants respond to maximum and minimum quantities of these resources is vital for designing suitable management approaches that improve crop performance and enhance resource use efficiency in resource-restricted situations (Teixeira et al., 2014). Water and nitrogen are the two main factors that play vital roles in the growth of turf grass and ornamental quality constraints. Especially in the early growing stages of turf grass, the amount of available water and nutrients determines the success or failure of turf establishment, time and quality. Nitrogen is an essential structural constituent of proteins, rubisco, nucleic acids, and chlorophyll in addition to some hormones, and its application in the form of fertilization is a vital agronomic management strategy to boost crop performance (Ata-Ul-Karim et al., 2016). In plants, nitrogen availability drives proper photosynthetic functional activity of the leaf (Brennan, 1992). In addition, effective nutrition levels have also alleviated drought stress damage by sustaining metabolic activities under reduced tissue water potential (Zhang et al., 2007). Hitherto, the response of Kentucky bluegrass to drought stress conditions during its vegetative growth phases has not been well understood. Water restrictions, along with reduced nitrogen application, are the key constraints on Kentucky bluegrass growth and development and have been broadly documented to influence the leaf water relations, chlorophyll fluorescence and photosynthetic traits, which results in reduced plant growth performance, early senescence, and diminished crop productivity (Park and Lee, 2003; Madani et al., 2010; Mobasser et al., 2014). An adequate evaluation of drought stress influence on the morpho-physiological traits under nitrogen fertilization can deliver valuable understanding of Kentucky bluegrass performance under drought stress (Teixeira et al., 2014; Abid et al., 2016).

*Poa pratensis* (scientific name: *P. pratensis* L.; English name: Kentucky bluegrass) belongs to Gramineae and is a perennial cold season rhizome – sparse clump grass with green leafcolour and attractive leaf and plant shapes. Strong vegetative propagation property of this grass will produce numerous ramets and form an uniform turf sod under desired water and nitrogen conditions. Ramets distributions in the grass clonal system connected by rhizomes largely rely on water and nitrogen status in soil (Eaton et al., 2004). Water and nutrient resources may differ significantly even at small spatial scales, because of environmental heterogeneity (Roiloa and Hutchings, 2012). In northern China arid and semi-arid regions, temperatures often approach 38°C or higher during the summer months, and water scarcity causes a high amount of soil nitrogen loss; therefore, drought stress and poor nutrient management are the two key limitations for the growth and turf uniformity of Kentucky bluegrass rate. Despite the availability of numerous studies concerning different species responses to drought stress, information is lacking on the effects of nitrogen and water variations in both homogeneous and heterogeneous environments with Kentucky bluegrass. Thus, the current research was conducted in North China to study the patterns of water and nitrogen sharing between ramets of Kentucky bluegrass and mechanism of morphological and physiological integration on water and nitrogen resources; and to investigate whether a nitrogen application increases the potential to withstand and recover from drought stress applied during Kentucky bluegrass growth periods. This study will be important for better understanding the adaptation of Kentucky bluegrass to different water and nutrition environment and to provide a theoretical basis for developing and improving drought-tolerant grass germplasm for environments with scarce water reserves.

## Materials and Methods

### Plant Material and Growth Conditions

Kentucky bluegrass ‘Arcadia’ provided by Shawn Bushman (USDA-ARS) was examined because it has been observed to perform well in field under scared water and nitrogen conditions (SRO, 2003). Grass seeds were sowed in plastic trays (70 cm × 30 cm × 10 cm) containing a soil matrix [peat soil: vermiculite: loam soil (ratio of 6: 3: 1)]. Before sowing, the soil matrix was first drenched, and after sowing, the seeds were covered with a thin layer of soil and placed in the dark for incubation. Germination began 5 days after sowing, and when the first two leaves were produced by seedlings, the seedlings were transplanted to pots (20-cm diameter and 40-cm height) filled with a matrix of peat soil, vermiculite and loam soil (a ratio of 6: 3: 1). After selecting healthy and similar size seedlings, plants were shifted to outdoor conditions (field) for further trials and observations. During this period, the average daily day and night temperature was 25 ± 2 and 15 ± 2°C, respectively. Relative humidity was 60 ± 5%, and natural sunlight at 700 ± 10 μmol m^-2^s^-1^ was maintained.

### Experimental Design

The field experiment was conducted in outdoor plots at the Northeast Agricultural University Horticulture experimental station. Sunny flat land with a cultivated heterogeneous habitat cell design was selected. The cells had a length and width of 60 cm and a depth of 50 cm. The bottom and sides of a cell were closed with polyethylene film to ensure that water and nutrients did not leach into separated areas, while the bottom was appropriately drilled to allow draining and prevent rot. The culture medium was sand excluding N, and with diameter 0.2 ∼ 2.0 mm, pH 7.0. Nutrition soil with total nitrogen content is 2.0% (Heilongjiang Richfield Company) was fertilized during the experiments.

**FIGURE 1:**
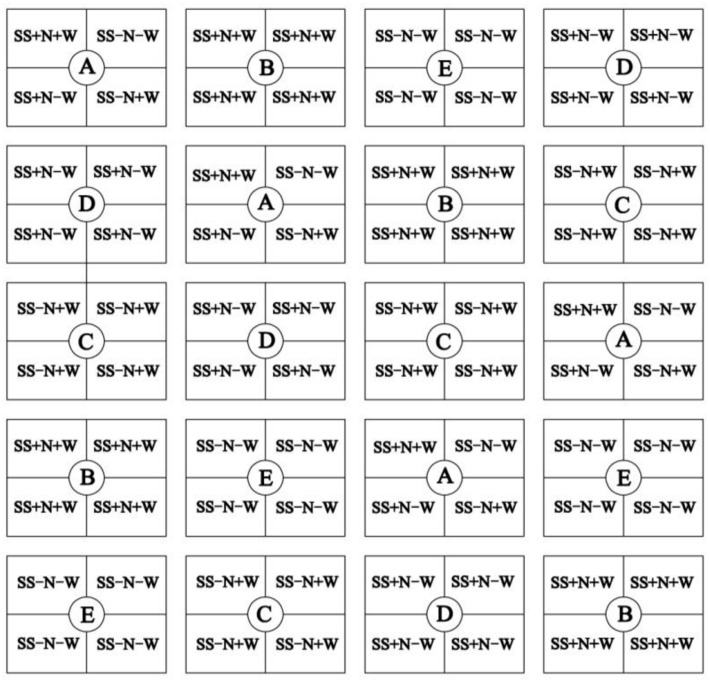
One heterogeneous habitat A (SS+N+W, SS–N+W, SS+N–W, SS–N–W) and four homogenous habitats B (SS+N+W), C (SS–N+W), D (SS+N–W), and E (SS–N–W). Each treatment had four replications. The symbols ‘+N’ and ‘+W’ indicates plots with full nitrogen or full water, while the ‘–N’ and ‘–W’ means plots without nitrogen or less water. All plots were arranged with randomized complete block design and were represented by this figure.

Different water conditions were applied after planting: one heterogeneous habitat A (SS+N+W, SS-N+W, SS+N-W, SS-N-W) and four homogenous habitats B (SS+N+W), C (SS-N+W), D (SS+N-W), and E (SS-N-W). Each treatment had four replications. The symbols ‘+N’ and ‘+W’ indicates plots with full nitrogen or water, while the ‘-N’ and ‘-W’ means plots without nitrogen or less water; [SS+N+W means sand with nutrient and full of water; SS-N+W means sand without nutrient but having water; SS+N-W means sand with nutrient but without water; SS-N-W means sand without both nutrient and water (**Figure 1**)]. Selected healthy bluegrass plants grown in each plot circle at a height of 5 cm were trimmed. After that, the four habitat treatments were completed in a precise way to study the changes in morphological growth and in the organizational structure of bluegrass ramets caused by these treatments. Sufficient irrigation water was supplied in the range of 8 L to habitats with full water treated every 2 days, while for drought stress, the same amount water was applied to the habitats with less water treated every 7 days. Nutritive soil was applied at the rate of 100 g/m^2^ to each cell once every after 10 days after planting during the trial. All plots were covered with plastic film to prevent rain water from entering into the field (Reserved vent), and when the rain stopped, the plastic was removed.

### Data Recorded

#### Nitrogen (N), Organic Carbon (C) and Total C to N Ratio (C:N) of Both Mother and Ramet Plants Measurements

For the determination of nitrogen (N) and organic carbon (C), leaf samples were ground after being dried at 80°C for 36 h and were passed through a 20 mesh screen. The total nitrogen (N) and organic carbon (C) content was determined by using the Semimicro-Kjeldahl method and the rapid dichromate oxidation technique, respectively (Nelson and Sommers, 1982). The total C to N ratio (C:N) (g g^-1^ DW) was calculated as an estimate for the long term nitrogen use efficiency (Livingston et al., 1999).

#### Above and Ground Mass (kg/plot) and Maximum Radius (cm), Number of Ramets Determinations

All bluegrass cells were dug up for biomass determination after measuring the necessary parameters. Genets (tiller, two and three point ramet strains) were classified separately, then rinsed with water and dried using filter paper. After drying at 85°C for 48 h in the oven, the above- and below ground biomass dry weight was measured. The maximum radius was also observed in Bluegrass genet tillers at 10 day intervals, which was measured visually using a ruler with the minimum scale of 0.01 cm. The number of ramets, was also determined in both plots of the experiment.

#### Leaf Relative Water Content (RWC) and Water Potential (Ψw) Measurements

The leaf RWC of fully expanded leaves was determined based on fresh (FW), turgid (TW), and dry weights (DW) using the following formula: RWC (%) = [(FW - DW)/(TW - DW)] × 100. Leaf fresh weight was instantly evaluated (Mettler AE260 balance, United States) after being removed from the plants and was then soaked in deionized water at room temperature 25 ± 1°C for 6 h. Next, for the determination of TW, all leaf samples were blotted dry and directly weighed. The samples were dried for DW measurements in an oven at 80°C for 72 h. The leaf water potential (Ψw) was determined following the method of Bruggink and Huang (1997) by using a pressure chamber (PMS Instrument Co., Corvallis, OR, United States). In the sealing sleeve of the specimen holder of the instrument, the leaves were firmly fixed and pressure was applied until the appearance of sap from the exposed end of the leaf was observed. At this stage, the reading was noted, which showed the negative force at which water was apprehended within the leaf and was expressed as -MPa.

#### Leaf Crosscutting Structure and SPAD Reading Determinations

For study of leaf crosscut structures, four leaves segments 3 mm long were cut from the middle of the second leaf from top in each treatment and were fixed in 2.5% glutaraldehyde (v/v) in 25 mol m^-3^ phosphate buffer at pH 7.0 (Huang and Fry, 1998), dehydrated in an ethanol series, critical-point dried with liquid CO_2_, and sputter-coated with gold-palladium. The xylem and phloem tissue, along with the sieve diameter and vascular circumference of the leaf segments were visually examined and photographed with the scanning electron microscope (S-4800, Japan) at 1000×. Ramets greenness (or relative chlorophyll content) was determined using a hand-held chlorophyll meter (measured as the optical density, SPAD reading, Minolta Camera Co., Osaka, Japan) in both homogenous and heterogeneous plots.

### Statistical Analyses

The experiment was carried out in randomized complete block design (RCBD) with four replicates. The data were analyzed by analysis of variance using SPSS v 9.0 software (SPSS, Inc., Chicago, IL, United States). The mean values were compared with the least significance difference test at the 0.05 probability level. Mean graphs were made by using Sigma plot v.10 (Systat Software, San Jose, CA, United States).

## Results

### Seasonal Changes of Nitrogen and Carbon Concentrations in the Mother Plants of Kentucky Bluegrass in Homogenous Treatments

**FIGURE 2:**
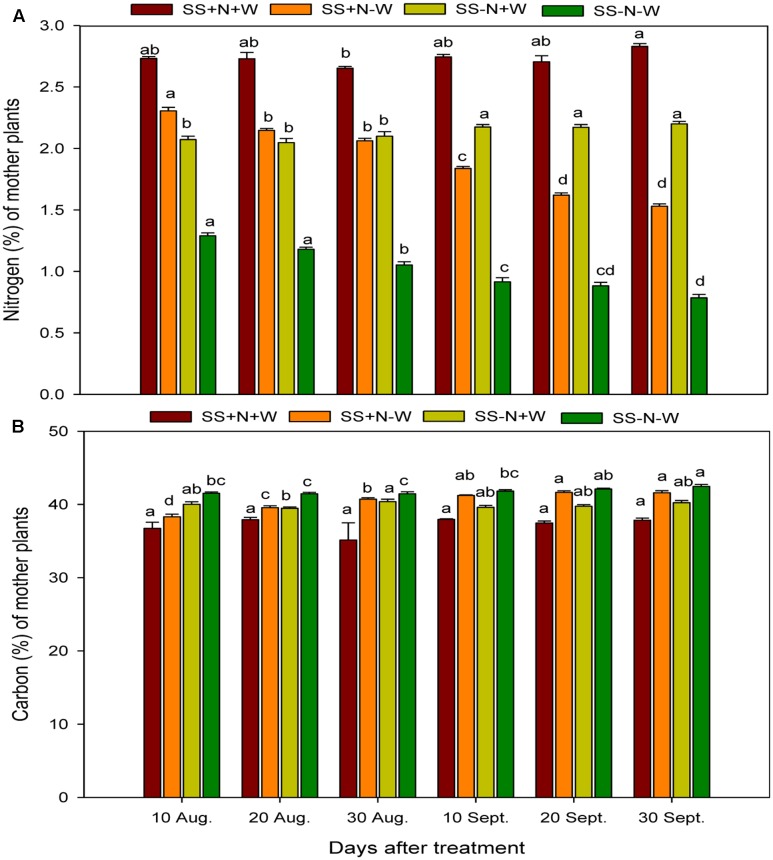
Seasonal changes of nitrogen and carbon concentrations in the mother plants of Kentucky bluegrass in homogenous plots. Error bars above the mean indicate the standard error (*n* = 4). Mean values for each treatment with different lower case letters indicate significant differences by the LSD-test (*P* < 0.05).

Nitrogen and carbon concentrations were severely hampered by drought stress in the mother plants of Kentucky bluegrass (**Figure 2A**). When plants were exposed to drought, nitrogen concentration gradually decreased in all treatment periods. Among the different day intervals, the nitrogen concentration on 30 September in the SS-N-W treatment rigorously decreased by 72%, followed by SS+N-W with a 45% decrease and SS-N+W with a 22% decrease compared to 10 August with the SS+N+W treatment. The drought treatment remarkably affected the nitrogen concentration; however, the use of nutritional soil enhanced the nitrogen concentration (49%) in the SS+N-W treatment compared to SS-N-W on 30 September. The carbon concentration was also significantly (*p* ≤ 0.05) influenced in the SS+N+W treatment with the mother plants at all durations compared to the rest of treatments. SS+N+W treatment reduced the carbon concentration (15%) on 30 August. Among all treatments, the effect of SS-N-W was more pronounced on carbon concentration, followed by SS+N-W, SS-N+W, and lastly SS+N+W at all days in the homogenous plot treatments (**Figure 2B**).

### Seasonal Changes of Nitrogen and Carbon Concentrations in the First Ramets of Kentucky Bluegrass in Homogenous Treatments

**FIGURE 3:**
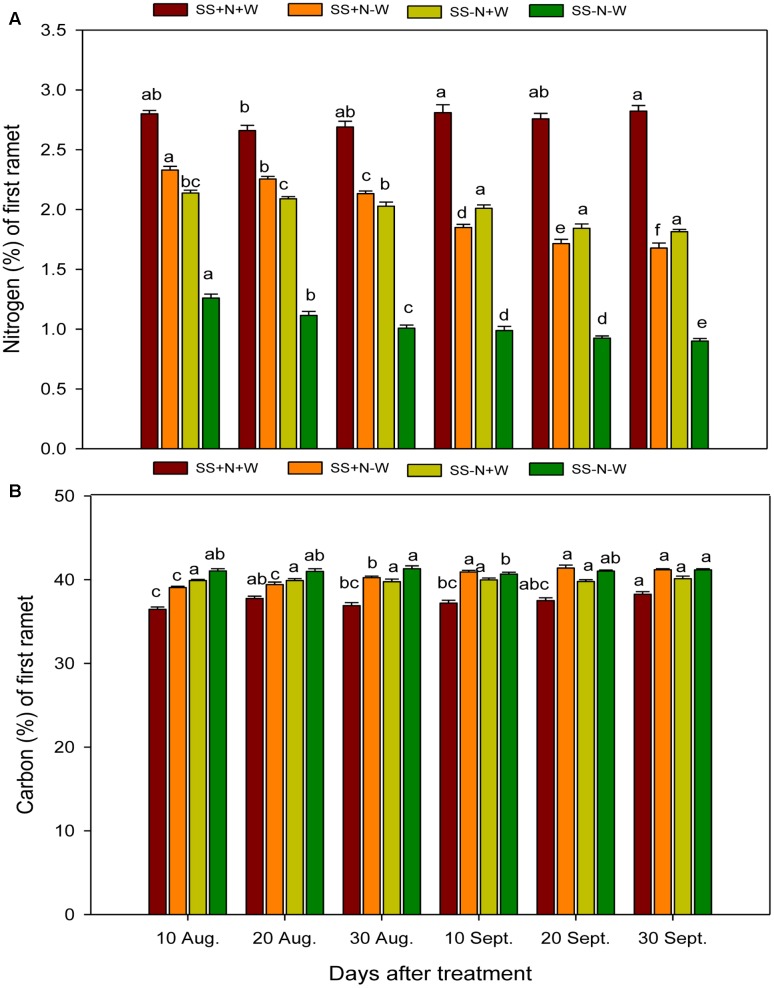
Seasonal changes of nitrogen and carbon concentrations in the first ramet of Kentucky bluegrass in homogenous plots. Error bars above the mean indicate the standard error (*n* = 4). Mean values for each treatment with different lower case letters indicate significant differences by the LSD-test (*P* < 0.05).

Data regarding to the nitrogen concentration in the first ramets of Kentucky bluegrass under influence of nutrition, well water and drought conditions are shown in **Figure 3A**. Drought stress was found to hamper the nitrogen concentration in the first ramets of Kentucky bluegrass plants at different time durations. The fertilization with nitrogen in nutrient soil remained beneficial, and SS+N+W recorded an increase of 36% in nitrogen compared to sandy soil without nitrogen SS-N+W on 30 September. On the other hand, the plants of SS+N-W also performed better than SS-N-W at all-time intervals. Applying the SS-N-W drought treatment after 30 September resulted in a 99% reduction in nitrogen concentration compared to the SS+N+W treatment. The influence of nutrition and sandy soil was significant (*p* ≤ 0.05) on carbon concentration during drought stress, as shown in **Figure 3B**. Among all treatments, SS+N-W and SS-N-W significantly enhanced carbon compared to SS+N+W at all-time interval durations. A maximum increase was recorded in the SS-N-W treatment (11%) on 30 September, followed by SS+N-W (10%) on 20 September compared to SS+N+W at 20 and 30 September, respectively. Carbon concentration was also enhanced in SS-N+W at all durations compared to the SS+N+W treatment in the first ramets of Kentucky bluegrass (**Figure 3B**).

### C:N Ratios of the Ramets and Mother Plants in Homogenous Plots

**FIGURE 4:**
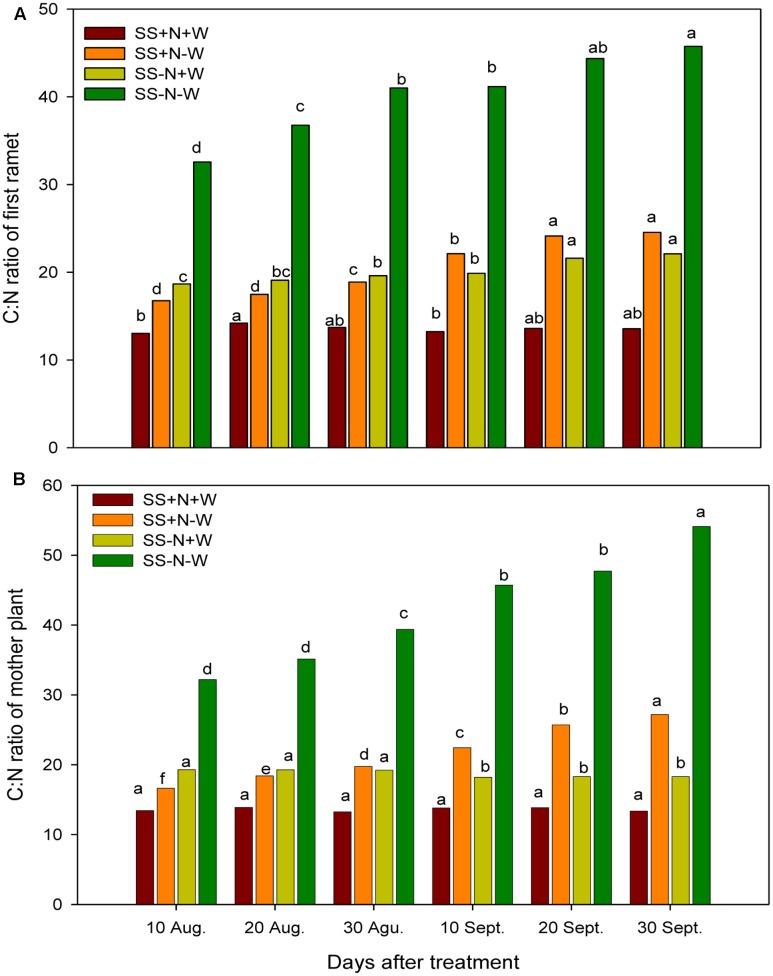
C:N ratios of the ramets and mother plants in homogenous plots. Mean values for each treatment with different lower case letters indicate significant differences by the LSD-test (*P* < 0.05).

The C:N ratio responded variably to the different treatments. A significant increase (*p* ≤ 0.05) was observed in the C:N ratio of both the ramets and mother plants of Kentucky bluegrass when plants were subjected to drought stress (**Figure 4**). The increase was more pronounced with the passage of time in drought treatments during the experiment. On 10 August and 30 September, the C:N ratios of the ramet plants were increased by 43 and 52% under drought stress in the SS-N-W treatment, respectively; however, the nutritional soil was more effective in decreasing (22 and 45% for 10 August and 30 September, respectively) the C:N ratio of the ramets in the SS+N-W treatment under stressful conditions. A similar trend was also observed in the mother plants in homogenous plots. With the induction of drought stress, an increase of 40 and 60% was recorded in the mother plants C:N ratios under the SS-N-W treatment on 10 August and 30 September. On other hand, SS+N-W treatment performed well in response to drought stress and reduced the C:N ratio up to 19 and 51% on 10 August and 30 September, respectively. Among all treatments, SS+N+W showed the lowest values of the C:N ratio throughout the experiment period.

### Effects of Different Water and Nutrient Supplies on the Maximum Radius (cm) of Kentucky Bluegrass at Different Day’s Intervals in Homogenous and Heterogeneous Plots (A)

**FIGURE 5:**
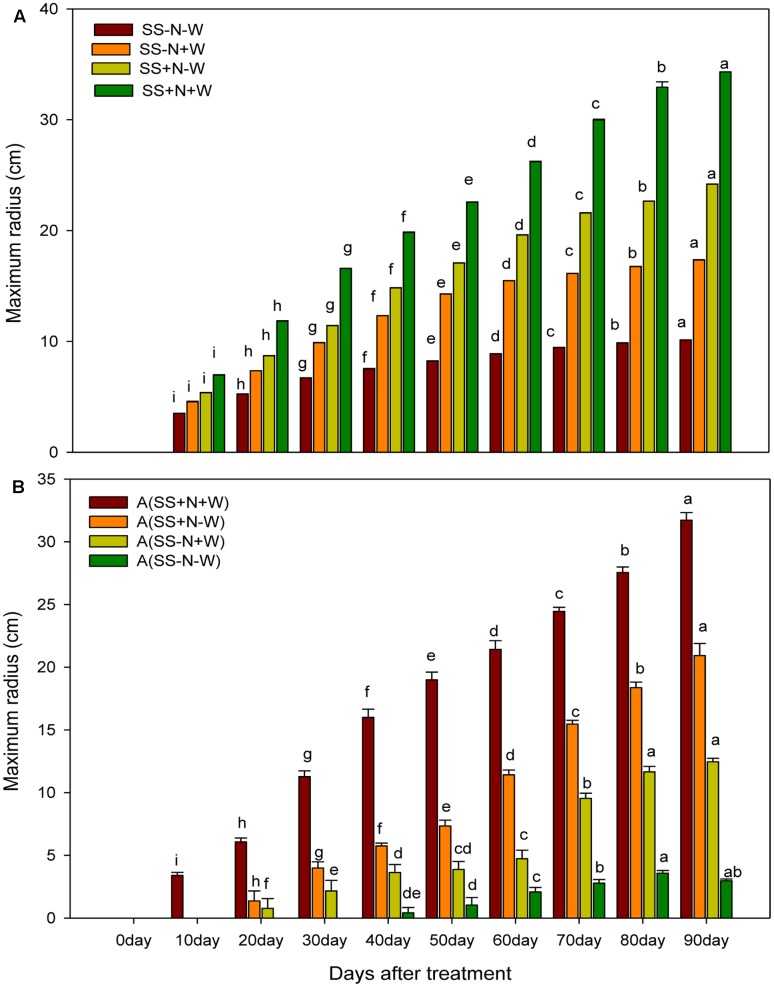
Effects of different water and nutrients supplies on the maximum radius (cm) of Kentucky bluegrass at different days interval in homogenous and heterogeneous (A) plots. Error bars above the mean indicate the standard error (*n* = 4). Mean values for each treatment with different lower case letters indicate significant differences by the LSD-test (*P* < 0.05).

Significant differences (*p* ≤ 0.05) among the different treatments were observed for the maximum radius of Kentucky blue grass in both homogenous and heterogeneous plots at different days. Across different well-watered and drought treatments, SS+N+W in homogenous plots and A (SS+N+W) in heterogeneous plots significantly influenced the maximum radius compared to the rest of the treatments (**Figures 5A,B**). The percent increase was less at the beginning of the experiment; 20 days after, rapid enhancement was observed in all well-watered treatments. In homogenous plots, drought stress severely influenced the maximum radius (70%) in the SS-N-W treatment at 90 days, while a minimum reduction of 50% at 10 days was also observed in the SS-N-W treatment compared to SS+N+W. Among all the other treatments, SS+N-W performed well, followed by SS+N-W and SS-N-W, as shown in **Figure 5B**. Similar trends were also observed in heterogeneous plots (A). In this plot, no data were recorded at 10 days for A (SS+N+W), while for A (SS-N+W) at 10 and 20 days, no data and data for only one replicate, respectively, were collected. For the A (SS-N-W) treatment, no data were collected for days 10, 20, and 30, while the data from two replicates at days 40 and 50 were not collected during the experiment. In this plot, drought stress severely reduced the maximum radius up to 91% in A (SS-N-W) at 90 days compared to A (SS+N+W). Sandy soil with well-watered treatment A (SS-N+W) showed an enhancement in the maximum radius (76%) compared to A (SS-N-W); however, in comparison with the A (SS+N-W) and A (SS+N+W) treatments, lower maximum radiuses (40 and 61%, respectively) were recorded.

### Effects of Different Water and Nutrient Supplies on the above and below Ground Biomass (kg/plot) of Kentucky Bluegrass in Homogenous and Heterogeneous Plots (A)

**FIGURE 6:**
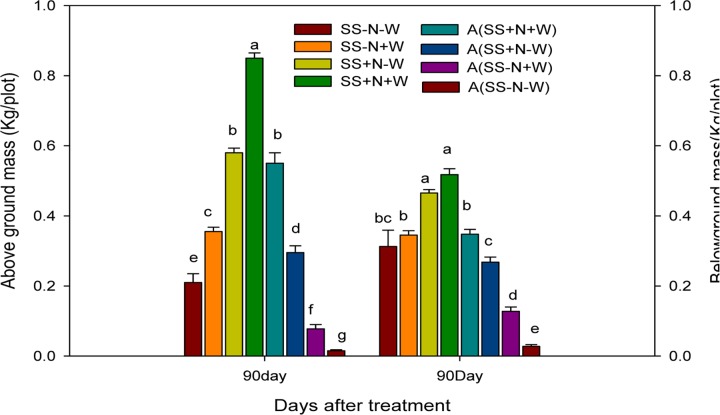
Effects of different water and nutrients supplies on the above and ground mass (Kg/plot) of Kentucky bluegrass in homogenous and heterogeneous (A) plots. Error bars above the mean indicate the standard error (*n* = 4). Mean values for each treatment with different lower case letters indicate significant differences by the LSD-test (*P* < 0.05).

Kentucky bluegrass growth was adversely influenced by drought conditions; however, well-watered and nutrition soil alleviated this severe effect. Maximum above and below ground biomass were recorded from Kentucky bluegrass raised under well-watered and nutrition soil conditions in both plots. In homogenous and heterogeneous plots, the SS-N-W treatments showed less than 75 and 40%, respectively, of above and below ground biomass at 90 days of treatment (**Figure 6**). Among all treatments, SS+N+W in homogenous and A (SS+N+W) in heterogeneous plots exhibited greater biomass, followed by SS+N-W and A (SS+N-W) when compared to the other treatments. Nutrition soil treatment SS+N-W and A (SS+N-W) in both plots sustainably enhanced the above and ground biomass under drought stress compared to the sandy soil SS-N-W and A (SS-N-W) treatments. Among both plots and treatments, greater above and below ground biomass of Kentucky bluegrass were observed in homogenous plots.

### Number of Ramets in Homogenous and Heterogeneous Plots (A)

**FIGURE 7:**
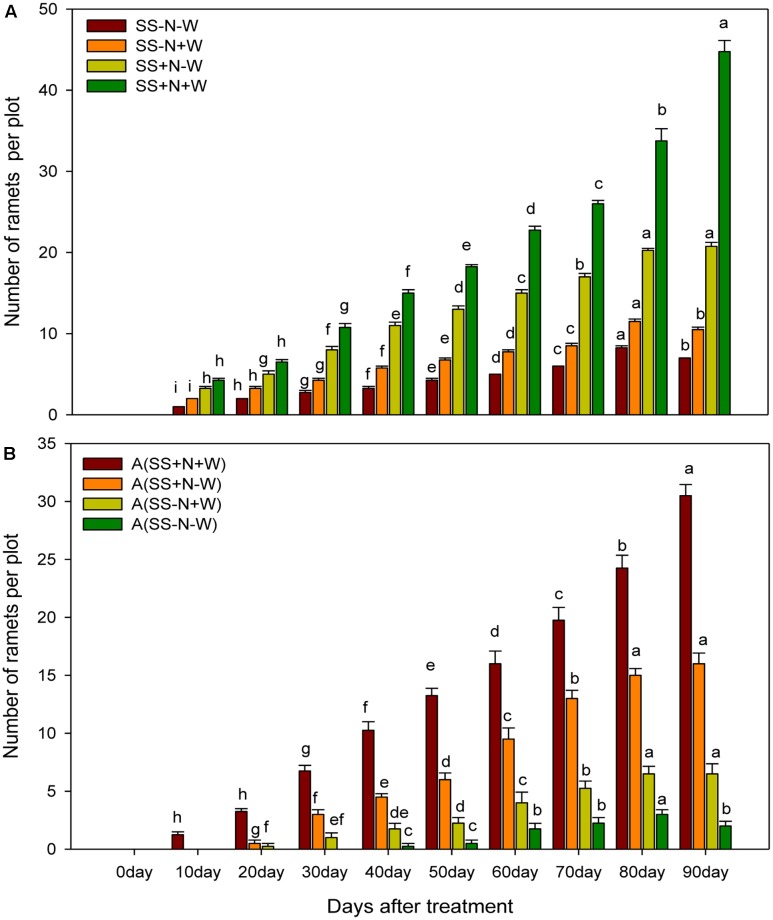
Effects of different water and nutrients supplies on number of the ramets in homogenous and heterogeneous (A) plots. Error bars above the mean indicate the standard error (*n* = 4). Mean values for each treatment with different lower case letters indicate significant differences by the LSD-test (*P* < 0.05).

Progressive water-deficient conditions significantly (*p* ≤ 0.05) affected ramet number in both homogenous and heterogeneous plots, as shown in **Figure 7A**. However, this reduction was more pronounced in the SS-N-W and A (SS-N-W) treatments. It was recorded that there were higher ramet numbers in SS+N-W and A (SS+N-W) with the progression of drought stress in both plots. Treatments SS+N+W and A (SS+N+W) showed 84 and 93% higher number of ramets, respectively, than the SS-N-W and A (SS-N-W) treatments at 90 days in both plots. Lower ramets numbers were observed at the start of the experiment (particularly at 10 and 20 days in all treatments); nevertheless, this number was enhanced with the passage of time, followed by a higher number at the end of experiment, particularly in nutrition soil treatments compared to sand soil (**Figure 7B**). Homogenous plots demonstrated higher ramet numbers compared to heterogeneous plots.

### Leaf Water Content (%) of Ramets in Homogenous and Heterogeneous Plots (A)

**FIGURE 8:**
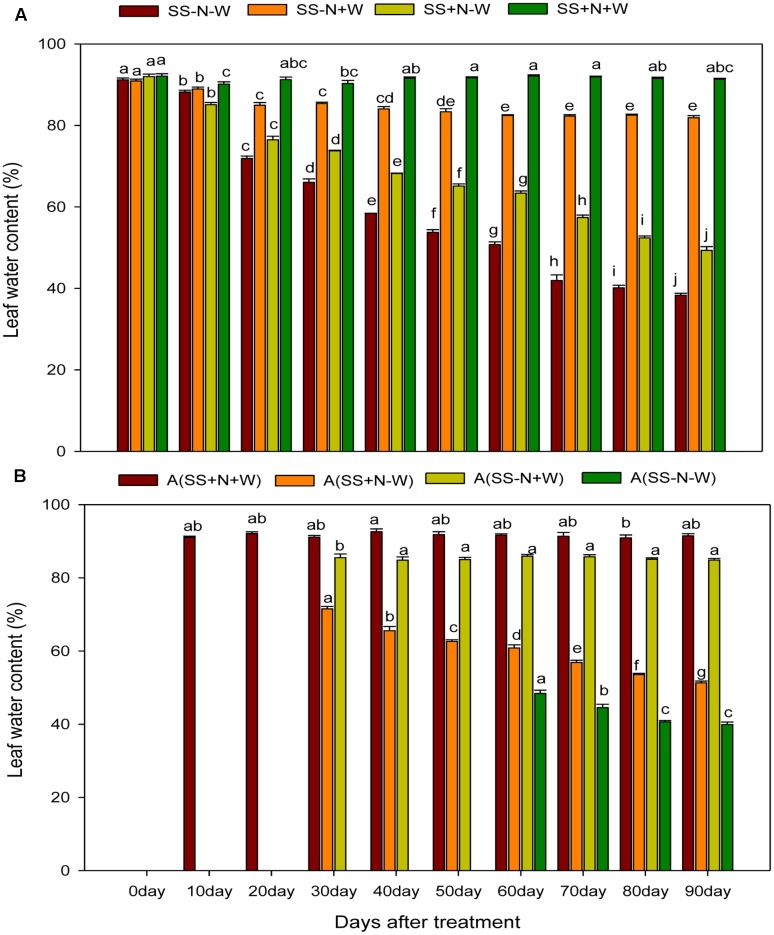
Influence of well-watered and deficit water conditions and nutrient supply on leaf water content (%) of the ramets in homogenous and heterogeneous (A) plots. Error bars above the mean indicate the standard error (*n* = 4). Mean values for each treatment with different lower case letters indicate significant differences by the LSD-test (*P* < 0.05).

The leaf water content of ramets were almost constant in both plots under well-watered and properly applied nutrient soil; however, this variable was significantly reduced in sandy soil and drought stress conditions during the experimental period. Higher leaf water content was observed at the start of the experiment, but with the passage time, it declined in drought treated plots. This decline was more pronounced in sandy soil compared to nutrition soil in both plots. In homogenous plots at 0 day, the SS-N-W treatment decreased only 0.28% for leaf water content; however, at 90 days, a severe reduction of 53% was observed compared to SS-N+W. On the other side, due to proper nutrient management, lower leaf water content (46%) in ramets reduced by drought stress was observed in SS+N-W compared to the SS+N+W treatment at 90 days (**Figure 8A**). In heterogeneous plots, data were not recorded for 0 day for all treatments, as shown in **Figure 8B**. Only A (SS+N+W) treatment data were observed at 10 and 20 days, while A (SS-N-W) treatment data were noted after 50 days during the experiment. Drought stress significantly reduced leaf water content by 53% in A (SS-N-W) at 90 days compared to the A (SS-N+W) treatment; however, only a 44% reduction was recorded in the A (SS+N-W) treatment than the A (SS+N+W) at 90 days of the experiment in heterogeneous plots.

### Leaf Water Potential (-Mpa) of Ramets in Homogenous and Heterogeneous Plots (A)

**FIGURE 9:**
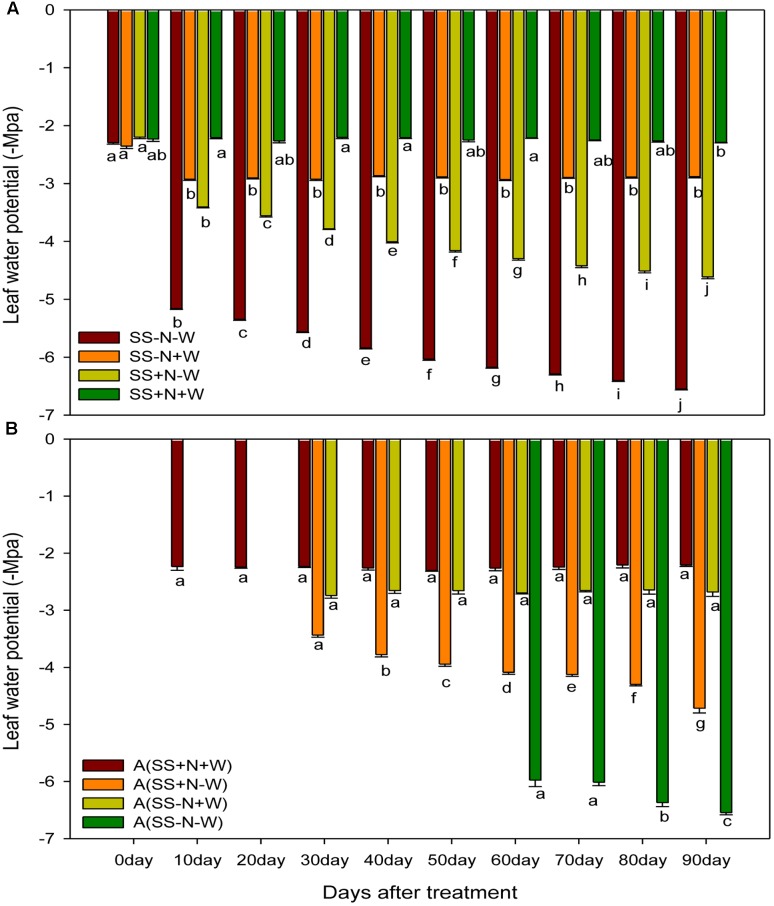
Influence of well-watered and deficit water conditions and nutrient supply on leaf water potential (–Mpa) of the ramets in homogenous and heterogeneous (A) plots. Error bars above the mean indicate the standard error (*n* = 4). Mean values for each treatment with different lower case letters indicate significant differences by the LSD-test (*P* < 0.05).

Drought stress progressively decreased the leaf water potential of ramets, while the highest leaf water potential was found in well-watered soil, which was maintained throughout the experimental period. Drought stress slightly reduced the leaf water potential in the early days, but with the passage of time, the severity of drought stress was more pronounced (**Figure 9A**). At 0 day of the experiment, the SS-N-W treatment did not significantly (*p* ≤ 0.05) influenced the leaf water potential, while at 90 days, the reduction was observed up to 56% compared to the SS-N+W treatment in homogenous plots. Similarly, in the SS+N-W treatment at 90 days, leaf water potential of ramets decreased 50% compared to SS+N+W; however, the severity of drought stress decreased by proper nutrient management in SS+N-W compared to SS-N-W. Again in heterogeneous plots, data were not taken for 0 day, while A (SS+N-W), A (SS-N+W) were not observed at 20 and 30 days. The data of A (SS-N-W) was taken from 60 to 90 days, as shown in **Figure 9B**. Similarly, drought stress decreased leaf water potential by 59% in A (SS-N-W) when compared with A (SS-N+W); however, this reduction was further enhanced (66%) compared with A (SS+N+W) at 90 days. Nutrition soil reduced the harmful effects of drought on the leaf water potential of the ramets to some extent during the study. A reduction of only 53% recorded in A (SS+N-W) grown ramets when compared with the A (SS+N+W) treatment at 90 days in heterogeneous plots.

### SPAD Readings of Ramets in Homogenous and Heterogeneous Plots (A)

**FIGURE 10:**
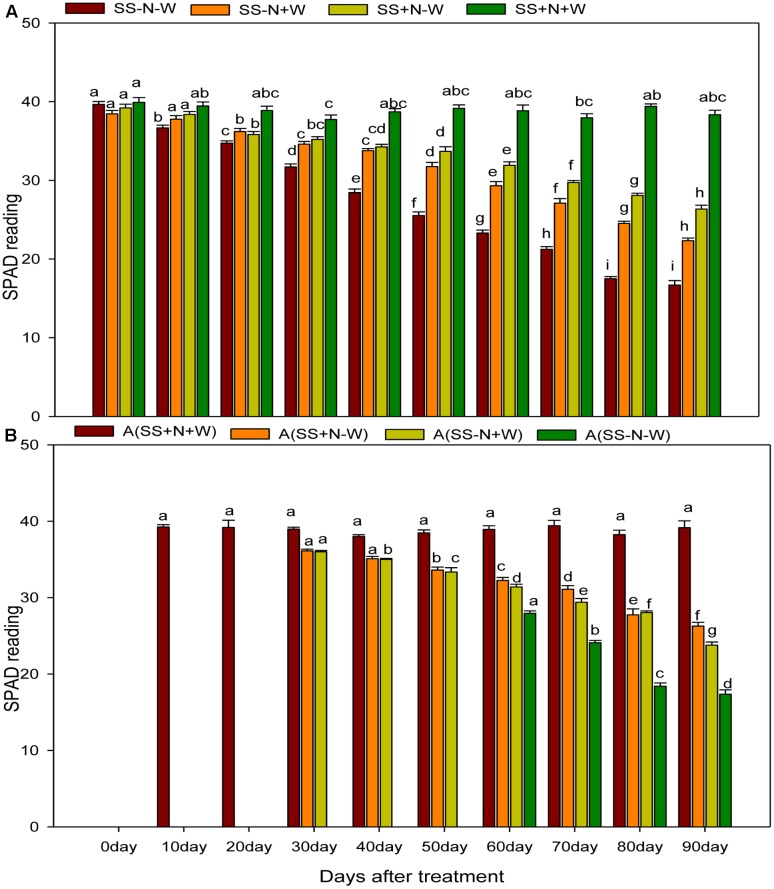
Influence of well-watered and deficit water conditions SPAD reading of the ramets in homogenous and heterogeneous (A) plots. Error bars above the mean indicate the standard error (*n* = 4). Mean values for each treatment with different lower case letters indicate significant differences by the LSD-test (*P* < 0.05).

Drought stress significantly (*p* ≤ 0.05) influenced SPAD values of Kentucky bluegrass ramets compared to well-watered and nutritional treatments (**Figure 10A**). When drought was induced, SPAD values progressively decreased with the passage of time during the experiment. Drought stress reduced SPAD values 1 and 56% in SS-N-W compared to the SS+N+W treatment at 0 and 90 days, respectively. However, when comparisons were made with SS-N+W with SS-N-W at 90 days, only a 25% difference in the SPAD value was observed between these two treatments. The effective use of nutritional soil ameliorated the adverse influence of drought on SPAD readings to some extent, and therefore, an enhancement of 37% was recorded in A (SS+N-W) at 90 days when compared with the A (SS-N-W) treatment in homogenous plots. Drought stress also led to a substantial SPAD value reduction in heterogeneous plots, as shown in **Figure 10B**. With the induction of drought stress, SPAD values decreased to 33% in the A (SS+N-W) treatment; however, this reduction (56%) was more severe in A (SS-N-W) compared to the A (SS+N+W) treatment at 90 days. Among all treatments, the highest SPAD readings were recorded in A (SS+N+W) and followed by A (SS+N-W).

### Changes of Leaf Tissue Structure of Clonal Plants of Kentucky Bluegrass in Homogeneous Habitats

**FIGURE 11:**
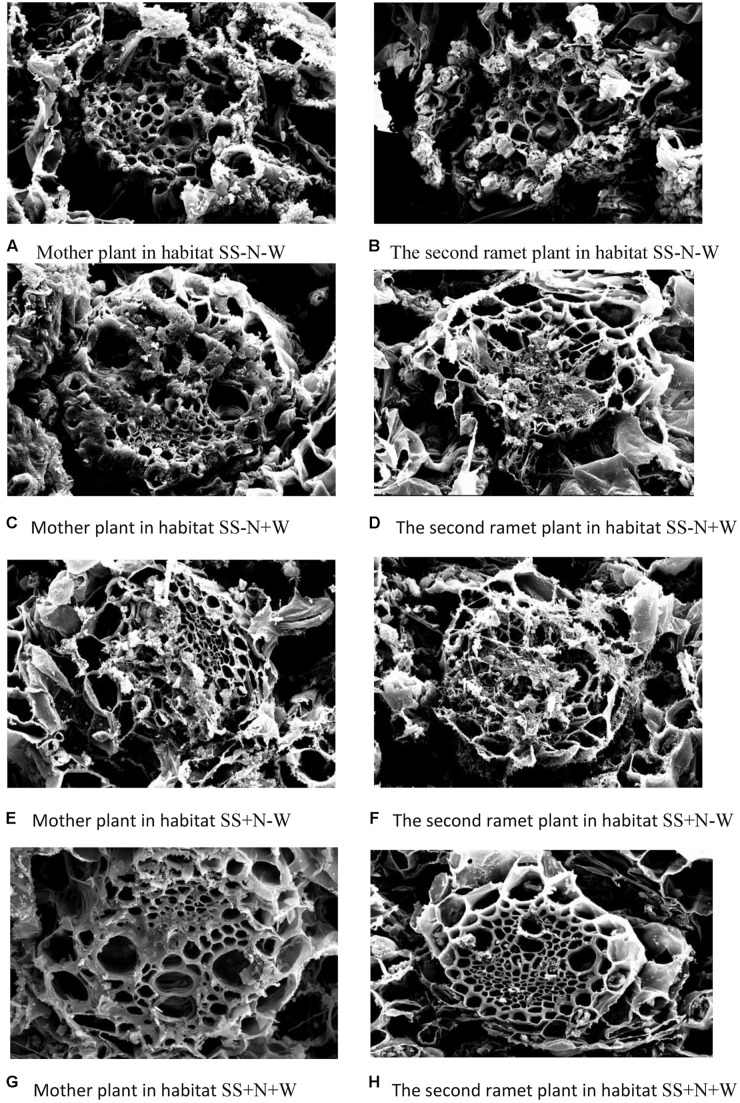
Electronic microscopic pictorial view of mother and remet plants of Kentucky bluegrass influenced by well-watered and deficit water conditions in homogenous plots.

The data in **Tables 1**–**4** and the pictorial view in **Figure 11** of the leaf crosscut structure of Kentucky bluegrass under 1000× using a transmission electron microscope show that the changes in vascular bundles of each index value of genet and clonal plants of Kentucky bluegrass in homogeneous habitats presents the same general trend. Each index value from high to low is as follows: genet, first ramet, second ramet, and third ramet. In the different habitats in the same class ramets, vessel diameter changed significantly from high to low as follows: C > B > E > D. Sieve diameter changed significantly (*p* ≤ 0.05) from large to small as follows: D > E > B > C. Vascular circumference changed significantly from high to low as follows: C > E > B > D. Phloem area changed significantly from large to small as follows: D > E > B > C. Xylem area changed significantly from high to low as follows: C > B > E > D. Under the habitats of the same culture medium and different water treatments, drought stress shrunk the vessel diameter and circumference, but increased the sieve diameter and xylem area. Under the habitats of the same water supply and different substrate conditions, vessel diameter and circumference were smaller in nutrition soil, while the sieve diameter and xylem areas were increased.

**Table 1:** Leaf crosscutting structure of Kentucky bluegrass under 1000 times the transmission electron microscope and analysis of the significance difference.

Homogeneous habitats	Xylem vessel diameter (μm)	Sieve diameter (μm)	Vascular circumference (μm)	Phloem area (μm^2^)	Xylem area (μm^2^)
B	13.15^b^	8.46^c^	192.44^bc^	261.75^bc^	441.33^b^
D	9.34^d^	11.69^a^	183.57^d^	284.52^a^	421.55^d^
C	16.46^a^	5.65^d^	207.65^a^	253.41^d^	465.74^a^
E	10.74^c^	9.78^b^	195.39^b^	265.64^b^	432.35^c^


*Different lower case letters of the same column indicate significant differences under different habitats (*p* < 0.05.). B indicates nutrition soil with full water; D shows nutrition soil with less water; C demonstrates sandy soil with full water; E indicates sandy soil with less water. The same is as follows.*

**Table 2:** The first ramet leaf crosscutting structure of Kentucky bluegrass under 1000 times the transmission electron microscope and analysis of the significance difference.

Homogeneous habitats	Xylem vessel diameter (μm)	Sieve diameter (μm)	Vascular circumference (μm)	Phloem area (μm^2^)	Xylem area (μm^2^)
B	11.76^b^	7.01^c^	160.35^bc^	224.66^bc^	387.52^b^
D	7.81^d^	10.22^a^	149.28^d^	243.75^a^	361.44^d^
C	15.02^a^	4.23^d^	172.83^a^	211.91^d^	403.85^a^
E	9.13^c^	8.29^b^	166.57^b^	229.77^b^	372.17^c^


**Table 3:** The second ramet leaf crosscutting structure of Kentucky bluegrass under 1000 times the transmission electron microscope and analysis of the significance difference.

Homogeneous habitats	Xylem vessel diameter (μm)	Sieve diameter (μm)	Vascular circumference (μm)	Phloem area (μm^2^)	Xylem area (μm^2^)
B	10.05^b^	6.25^c^	142.33^b^	190.33^bc^	325.77^b^
D	7.02^d^	9.31^a^	109.11^d^	215.72^a^	301.28^d^
C	13.73^a^	3.72^d^	149.22^a^	180.51^d^	342.85^a^
E	8.16^c^	7.24^b^	139.55^bc^	197.64^b^	316.44^c^


**Table 4:** The third ramet leaf crosscutting structure of Kentucky bluegrass under 1000 times the transmission electron microscope and analysis of the significance difference.

Homogeneous habitats	Xylem vessel diameter (μm)	Sieve diameter (μm)	Vascular circumference (μm)	Phloem area (μm^2^)	Xylem area (μm^2^)
B	9.17^b^	5.35^c^	122.35^bc^	165.17^bc^	273.58^b^
D	6.12^d^	8.47^a^	114.56^d^	183.52^a^	244.65^d^
C	11.95^a^	3.06^d^	134.18^a^	157.29^d^	283.22^a^
E	7.86^c^	6.58^b^	126.57^b^	168.77^b^	259.79^c^


## Discussion

Climate change is one of the most detrimental challenges that cause serious concerns to agriculture sector mostly in climate-sensitive developing countries of the world. Due to climatic variations, the damaging influences of water scarcity are not only bounded to food security but have an extensive consequences on other sector of agriculture production systems. The drought intensity is unpredictable as it depends on numerous issues like rainfall occurrence and their distribution, evaporative demands and soils capability of preserving moisture contents. Hence, proper planning for sustainable water usage and improvement of drought resistant cultivars is imperative (Chaves et al., 2003). The plant response mechanism to water scarcity condition is extremely intricate which varies among plant types and growth duration along with the period of water restriction (Farooq et al., 2009). In reality, lower crop water requirements in temperate climatic regions and more irrigation for crops under arid regions are subjected to incessant series of drought stress and re-watering (Perrone et al., 2012). Lately, more attention has been given to plants’ capacity for drought recovery (Chaves et al., 2009; Luo, 2010; Perrone et al., 2012; Vanková et al., 2012; Fang and Xiong, 2015). Little is known about the plants clonal system reaction with the varied nutrition and water environments. Actually in nature, the necessity resources for plants growth and production such as sunlight, water, or soil nutrients were heterogeneous distribution in space and time. The patchy scatter of nitrogen and water in soil largely determines the ramets of Kentucky bluegrass distribution and uniformity which is one of the important ornamental and functional characteristics on turf. In this research, the response of nutrition and water on Kentucky bluegrass were comparatively analyzed for adaptation to drought stress. Drought stress severely hampered all morphological attributes, nitrogen utilization, carbon content and leaf structure of Kentucky bluegrass; nevertheless, the use of nutritional soil was helpful to a large extent in ameliorating the adverse influence of drought stress. With the induction of drought stress, the C:N ratios of Kentucky bluegrass in both mother plants and ramets were enhanced during the study because of a reduction in N and increased levels of C content (**Figures 2**–**4**). Less water availability under drought stress normally decreases the uptake of total nutrients and lowers tissue concentrations in plants. Additionally, such influences may also be associated with restricted energy accessibility for NO_3_^-^/NH_4_^+^ under water limited conditions (Farooq et al., 2009). Inside the plant when the water level falls below a critical point, as a result stomata closure occur which causes a reduction in transpiration rate and therefore restrict the water transportation to the plant. Consecutively, influences the roots capability to uptake water and nutrients as efficiently as pretend to be under normal transpiration (Waraich et al., 2011).

Numerous studies have documented that drought stress influences carbon metabolism, and therefore, in several species, carbon is enhanced in roots and becomes acclimatized to soil water deficits; this was also verified from the results during our study (Assuero et al., 2002). Broadley et al. (2000) also observed a strong correlation between C assimilation and leaf N in lettuce plant. For better plant production nitrogen is considered one of the important nutrients. Our results indicated that drought stress decreased nitrogen concentration; this was also confirmed by De Souza et al. (1997) who reported that the reduction of leaf nitrogen and chlorophyll content by drought stress enhanced leaf senescence of the plants. Shangguan et al. (2000) observed that drought stress reduced nitrogen concentration in the leaf by 40.3% for a low level of N and was 25.4% for the high level of N treatments on the 9th day.

Decreasing morphological characters such as radius, above and ground biomass and the number of ramets under water stress might be due to the reduction in RWC that caused the withering of leaves and thus affected these attributes (**Figures 5**–**7**). In the present study, the maximum radius, above and ground biomass, and the number of ramets in both homogenous and heterogeneous plots decreased under drought stress at different durations of time. Our results were further confirmed by Liu et al. (2008) who also observed that the quality of Kentucky bluegrass leaves were reduced in soil that faced a shortage of water. Similarly, in sunflower and *Catharanthus roseus* plants, increased root growth was noticed in response to drought stress (Tahir et al., 2002; Jaleel et al., 2008). Several earlier authors have connected such enhancement in the root/shoot ratio to the ABA content of roots and shoots under water deficient conditions (Sharp and Lenoble, 2002; Manivannan et al., 2007). Water losses through abnormal transpiration caused by drought conditions induces injury and cell desiccation, resulting in the reduction the turf quality and RWC and enhancing electrolyte leakage (Abraham et al., 2004). Our results are further confirmed by Arun et al. (2012) and Binghua et al. (2012) who showed that with an application of nitrogen, plants show positive influence in terms of growth and development under drought stress. Plant responses to N applications and soil water recommend that an N application could positively influence better soil moisture and plant growth (Song et al., 2010). Therefore, from these results, we can note that an N application could play an important role in mitigating the negative influences of water stress on the growth and development of plants. Therefore, using nutritional soil, the morphological attributes such as radius, above and ground biomass and the number of ramets under water had returned to 70% of the pre-stress level during the study. Therefore, our results recommend that nutritional soil can assure recovery potential once drought stress is relieved.

For maintaining perfect physiological functions and growth, water status played a pivotal role. Numerous researchers have documented that high RWC is closely associated with deficient water stress resistance (Altinkut et al., 2001; Keles and Oncel, 2004). In the current research, leaf water content and leaf water potential were severely diminished by drought stress; however, with the use of proper nutrient soil, enhancements were recorded in these parameters. Such reductions could be due to lower water availability to the root systems and compensating for water loss by the processes of transpiration (**Figures 8**, **9**). Egilla et al. (2005) noticed that in *Hibiscus rosa-sinensis*, water stress severely reduced *relative* water content, stomatal conductance, turgor potential, transpiration, and water use efficiency. Siddique et al. (2001) showed that the concomitant enhancement in leaf temperature with the imposition of water stress considerably reduced the RWC, leaf water potential, and transpiration rate in wheat plants. Therefore, these results revealed that leaf water potential may play a role in indicating the water status of a plant and shows the capability of plants to sustain maximum water, which develops better adaptability to drought by increasing tolerance to drought stress but not to drought recovery. Root system also play a vital role in maintaining better RWC and leaf water potential of the plants. By altering the allocation design, water absorption is maximized namely augmenting investments in the roots. Instead of absorbing water actively, roots just let it to pass through them in reaction to water potential gradients generally set up by transpiration. The intricate roots anatomical assembly results in a multifaceted form of water flow. Transport through numerous tissues like epidermis, cortex, stele etc. has to be interpretive during the radial channel through the root cylinder along with a longitudinal flow constituent (axial transport in xylem vessels). Modification occurs in the anatomy of root tissue under stressful conditions, mainly, because stress (such as drought stress) encourages the progress of apoplastic blockades for water and ion movement (Taleisnik et al., 1999). This effects the balance of water by decreasing the roots ability to up take water.

SPAD readings were significantly influenced by the use of nutritional soil under drought stress, and this could be due to a better uptake of water, root growth and uptake, root growth, and leaf erectness (Gong et al., 2003; Hattori et al., 2005) that was retained with higher leaf water potential and leaf green color in the current research (**Figure 10**). Drought stress can diminish the chlorophyll content and prevent its further production (Lessani and Mojtahedi, 2002). Numerous studies have accounted for injury to the leaf pigments as a consequence of drought stress (Nilsen and Orcutt, 1996; Montagu and Woo, 1999). A four unit enhancement was observed by Dhanda et al. (2004) in SPAD readings when the RWC was reduced from 94 to 87%; nevertheless, there are many studies documenting the negative influences of drought stress on leaf chlorophyll content (Ashraf et al., 1994; Sairam et al., 1997). Chlorophyll concentration and leaf color are directly correlated with nitrogen content. Due to drought conditions and nitrogen deficiency, SPAD readings were decreased by drought stress in sandy soil compared to nutritional soil, and our results were also verified by Carroll et al. (2015) who also observed the same trend during his research.

Drought stress also influenced the leaf tissue structure of clonal plants of Kentucky bluegrass in homogeneous habitats. In agreement with earlier studies (Gironde et al., 2015), a decrease in number of leaf ranks was recorded in stressed plants, related to an interruption of plant development (**Tables 1**–**4** and **Figure 11**) (Gombert et al., 2006; Masclaux-Daubresse et al., 2008; Albert et al., 2012). The responses of Kentucky bluegrass to environmental stress conditions and the depletion of a nitrogen application may also have influenced the growth of young leaves, as previously accounted for in the literature (Bouchet et al., 2014).

## Conclusion

Water stress is the most damaging environmental setback for plant performance. To the best of our knowledge, information is lacking on the effects of nitrogen and water variations in both homogeneous and heterogeneous environments with Kentucky bluegrass. Conclusively, our results demonstrated that drought stress severely influenced all agronomical, anatomical and physiological attributes of Kentucky bluegrass in both homogenous and heterogeneous plots. However, use of nutritional soil with a proper nitrogen rate remained effective in ameliorating the adverse influence of drought stress. Considering the results of the present study collectively, the better performance of nutritional soil under stressful conditions seems to arise from (1) enhanced RWC and leaf water potential, (2) a reduction in the C:N ratio, (3) increased agronomical and physiological attributes, and (4) a better leaf tissue structure of the clonal plants. Our study provides an insight and is a step closer in ascertaining the role of nutritional soil for a better performance of water-stressed Kentucky bluegrass. From these results, we recommend that this research might help to understand the growth and development characteristics and adaptive strategies of Kentucky bluegrass under drought conditions, and theoretically and practically guide the scientific management of Kentucky bluegrass.

## Author Contributions

SS and CY initiated and designed the research. SS performed the experiments. SS and CY and analyzed the data and wrote the manuscript SF, MI, HH, WN, AU, MA, HA, and CY revised and edited the manuscript and also provided advice on the experiments.

## Conflict of Interest Statement

The authors declare that the research was conducted in the absence of any commercial or financial relationships that could be construed as a potential conflict of interest.

## References

[B1] Abid M. Tian Z. Ata-Ul-Karim S. T. Cui Y. Liu Y. Zahoor R. (2016). Nitrogen nutrition improves the potential of wheat (*Triticum aestivum* L.) to alleviate the effects of drought stress during vegetative growth periods. *Front. Plant Sci.* 7:981 10.3389/fpls.2016.00981 PMC4927619 27446197

[B2] Abraham E. M. Huang B. Bonos S. A. Meyer W. A. (2004). Evaluation of drought resistance for texas bluegrass, kentucky bluegrass, and their hybrids. *Crop Sci.* 44 1746–1753. 10.2135/cropsci2004.1746

[B3] Albert B. Le Caherec F. Niogret M. F. Faes P. Avice J. C. Leport L. (2012). Nitrogen availability impacts oilseed rape (*Brassica napus* L.) plant water status and proline production efficiency under water-limited conditions. *Planta* 236 659–676. 10.1007/s00425-012-1636-8 22526495 PMC3404282

[B4] Altinkut A. Kazan K. Ipekci Z. Gozukirmizi N. (2001). Tolerance to paraquat is correlated with the traits associated with water stress tolerance in segregating F2 populations of barley and wheat. *Euphytica* 121 81–86. 10.1023/A:1012067711200

[B5] Anjum S. A. Xie X. Y. Wang L. C. Saleem M. F. Man C. Lei C. W. (2011). Morphological, physiological and biochemical responses of plants to drought stress. *Afr. J. Agric. Res.* 6 2026–2032.

[B6] Arun T. Upadhyaya S. D. Upadhyay A. Preeti Sagar N. (2012). Responses of moisture stress on growth, yield and quality of isabgol (*Plantago ovata* Forsk). *J. Agric. Technol.* 8 563–570.

[B7] Ashraf M. Y. Azmi A. R. Khan A. H. Ala S. A. (1994). Effect of water stress on total phenols, peroxidase activity and chlorophyll content in wheat. *Acta Physiol. Plant.* 16 185–191.

[B8] Aslam M. Zamir M. S. I. Anjum S. A. Khan I. Tanveer M. (2014). An investigation into morphological and physiological approaches to screen maize (*Zea mays* L.) cultivars for drought tolerance. *Cereal Res. Commun.* 43 41–51. 10.1556/CRC.2014.0022

[B9] Assuero S. G. Matthew C. Kemp P. Barker D. J. (2002). Effects of water deficit on mediterranean and temperate cultivars of tall fescue. *Crop Pasture Sci.* 53 29–40. 10.1071/AR01023

[B10] Ata-Ul-Karim S. T. Liu X. Lu Z. Yuan Z. Zhu Y. Cao W. (2016). In- season estimation of rice grain yield using critical nitrogen dilution curve. *Field Crops Res.* 195 1–8. 10.1016/j.fcr.2016.04.027

[B11] Binghua L. Liang C. Mingjun L. Dong L. Yangjun Z. Fengwang M. (2012). Interactive effects of water and nitrogen supply on growth, biomass partitioning, and water-use efficiency of young apple trees. *Afr. J. Agric. Res.* 7 978–985. 10.5897/ajar11.1212

[B12] Bouchet A.-S. Nesi N. Bissuel C. Bregeon M. Lariepe A. Navier H. (2014). Genetic control of yield and yield components in winter oilseed rape (*Brassica napus* L.) grown under nitrogen limitation. *Euphytica* 199 183–205. 10.1007/s10681-014-1130-4

[B13] Brennan R. F. (1992). The role of manganese and nitrogen nutrition in the susceptibility of wheat plants to take-all in Western Australia. *Fertil. Res.* 31 35–41. 10.1007/BF01064225

[B14] Broadley M. R. Escobar-Gutierrez A. J. Burns A. Burns I. G. (2000). What are the effects of nitrogen deficiency on growth components of lettuce? *New Phytol.* 147 519–526. 10.1046/j.1469-8137.2000.00715.x 33862945

[B15] Bruggink E. Huang C. (1997). Vessel contents of leaves after excision: a test of the scholander assumption. *Am. J. Bot.* 84 1217–1222. 10.2307/2446045 21708676

[B16] Carroll A. Lindsey C. Baker J. Hopkins B. G. Hansen N. (2015). “Drought and nitrogen stress effects on maize canopy temperature,” in *Proceedings of the Western Nutrient Management Conference* Vol. 11 Reno, NV, 84–89.

[B17] Chaves M. M. Flexas J. Pinheiro C. (2009). Photosynthesis under drought and salt stress: regulation mechanisms from whole plant to cell. *Ann. Bot.* 103 551–560. 10.1093/aob/mcn125 18662937 PMC2707345

[B18] Chaves M. M. Maroco J. P. Pereira J. S. (2003). Understanding plant responses to drought from genes to the whole plant. *Funct. Plant Biol.* 30 239–264. 10.1071/FP02076 32689007

[B19] Dai A. (2013). Increasing drought under global warming in observations and models. *Nat. Clim. Change* 3 52–58. 10.1038/NCLIMATE1811

[B20] De Souza P. I. Egli D. B. Bruening W. P. (1997). Water stress during seed filling and leaf senescence in soybean. *Agron. J.* 89 807–812. 10.2134/agronj1997.00021962008900050015x

[B21] Dhanda S. S. Sethi G. S. Behl R. K. (2004). Indices of drought tolerance in wheat genotypes at early stages of plant growth. *J. Agron. Crop Sci.* 190 6–12. 10.1111/j.1439-037X.2004.00592.x

[B22] Eaton T. D. Curley J. Wiliamson R. C. Jung G. (2004). Determination of the level of variation in polyploidy among Kentucky bluegrass cultivars by means of flow cytometry. *Crop Sci.* 4 2168–2174. 10.2135/cropsci2004.2168

[B23] Egilla J. N. Davies F. T. Jr. Boutton T. W. (2005). Drought stress influences leaf water content, photosynthesis, and water-use efficiency of *Hibiscus rosa-sinensis* at three potassium concentrations. *Photosynthetica* 43 135–140. 10.1007/s11099-005-5140-2

[B24] Fahad S. Bano A. (2012). Effect of salicylic acid on physiological and biochemical characterization of maize grown in saline area. *Pak. J. Bot.* 44 1433–1438.

[B25] Fahad S. Nie L. Chen Y. Wu C. Xiong D. Saud S. (2015). “Crop plant hormones and environmental stress,” in *Sustainable Agriculture Reviews*, Vol. 15 ed. Lichtfouse E. (Geneva: Springer International Publishing), 371–400. 10.1007/978-3-319-09132-7_10

[B26] Fang Y. Xiong L. (2015). General mechanisms of drought response and their application in drought resistance improvement in plants. *Cell. Mol. Life Sci.* 72 673–689. 10.1007/s00018-014-1767-0 25336153 PMC11113132

[B27] Farooq M. Wahid A. Kobayashi N. Fujita D. Basra S. M. A. (2009). Plant drought stress: effects, mechanisms and management. *Agron. Sustain. Dev.* 29 185–212. 10.1051/agro:2008021

[B28] Fischlin A. Midgley G. F. Price J. T. Leemans R. Gopal B. Turley C. (2007). “Ecosystems, their properties, goods, and services. Climate change 2007: impacts, adaptation and vulnerability,” in *Proceedings of the Contribution of Working Group II to the Fourth Assessment Report of the Intergovernmental Panel on Climate Change*, eds Parry M. L. Canziani O. F. Palutikof J. P. Hanson C. E. (Cambridge: Cambridge University Press), 211–272.

[B29] Fry J. Huang B. (2004). *Applied Turfgrass Science and Physiology.* Hoboken, NJ: Wiley.

[B30] Gironde A. Poret M. Etienne P. Trouverie J. Bouchereau A. Le Caherec F. (2015). A profiling approach of the natural variability of foliar N remobilization at the rosette stage gives clues to understand the limiting processes involved in the low N use efficiency of winter oilseed rape. *J. Exp. Bot.* 66 2461–2473. 10.1093/jxb/erv031 25792758

[B31] Gombert J. Etienne P. Ourry A. Le Dily F. (2006). The expression patterns of SAG12/Cab genes reveal the spatial and temporal progression of leaf senescence in *Brassica napus* L. with sensitivity to the environment. *J. Exp. Bot.* 57 1949–1956. 10.1093/jxb/erj142 16720615

[B32] Gong H. Chen K. Chen G. Wang S. Zhang C. (2003). Effects of silicon on growth of wheat under drought. *J. Plant Nutr.* 26 1055–1063. 10.1081/PLN-120020075

[B33] Hattori T. Inanaga S. Araki H. An P. Morita S. Luxová M. (2005). Application of silicon enhanced drought tolerance in *Sorghum bicolor*. *Physiol. Plant.* 123 459–466. 10.1111/j.1399-3054.2005.00481.x

[B34] Huang B. Fry J. D. (1998). Root anatomical, physiological, and morphological responses to drought stress for tall fescue cultivars. *Crop Sci.* 38 1017–1022. 10.2135/cropsci1998.0011183X003800040022x

[B35] Jackson R. B. Sperry J. S. Dawson T. E. (2000). Root water uptake and transport: using physiological processes in global predictions. *Trends Plant Sci.* 5 482–488. 10.1016/S1360-1385(00)01766-0 11077257

[B36] Jaleel C. A. Manivannan P. Lakshmanan G. M. A. Gomathinayagam M. Panneerselvam R. (2008). Alterations in morphological parameters and photosynthetic pigment responses of *Catharanthus roseus* under soil water deficits. *Colloids Surf. B Biointerfaces* 61 298–303. 10.1016/j.colsurfb.2007.09.008 17949951

[B37] Jaleel C. A. Manivannan P. Wahid A. Farooq M. Somasundaram R. Panneerselvam R. (2009). Drought stress in plants: a review on morphological characteristics and pigments composition. *Int. J. Agric. Biol.* 11 100–105.

[B38] Keles Y. Oncel I. (2004). Growth and solute composition in two wheat species experiencing combined influence of stress conditions. *Russ. J. Plant Physiol.* 51 203–208. 10.1023/B:RUPP.0000019215.20500.6e

[B39] Kusaka M. Ohta M. Fujimura T. (2005). Contribution of inorganic components to osmotic adjustment and leaf folding for drought tolerance in pearl millet. *Physiol. Plant.* 125 474–489. 10.1111/j.1399-3054.2005.00578.x

[B40] Lessani H. Mojtahedi M. (2002). *Introduction to Plant Physiology*, 6th Edn Tehran: Tehran University press.

[B41] Liu J. Xie X. Du J. Sun J. Bai X. (2008). Effects of simultaneous drought and heat stress on Kentucky bluegrass. *Sci. Hortic.* 115 190–195. 10.1016/j.scienta.2007.08.003

[B42] Livingston N. J. Guy R. D. Sun Z. J. Ethier G. J. (1999). The effects of nitrogen stress on the stable carbon isotope composition, productivity and water use efficiency of white spruce (*Picea glauca* (Moench) Voss) seedlings. *Plant Cell Environ.* 22 281–289. 10.1046/j.1365-3040.1999.00400.x

[B43] Luo L. J. (2010). Breeding for water-saving and drought-resistance rice (WDR) in China. *J. Exp. Bot.* 61 3509–3517. 10.1093/jxb/erq185 20603281

[B44] Madani A. Rad A. S. Pazoki A. Nourmohammadi G. (2010). Wheat (*Triticum aestivum* L.) grain filling and dry matter partitioning responses to source: sink modifications under post anthesis water and nitrogen deficiency. *Acta Sci. Agron.* 32 145–151. 10.4025/actasciagron.v32i1.6273

[B45] Manivannan P. Jaleel C. A. Sankar B. Kishorekumar A. Somasundaram R. Lakshmanan G. M. (2007). Growth, biochemical modifications and proline metabolism in *Helianthus annuus* L. as induced by drought stress. *Colloids Surf. B Biointerfaces* 59 141–149. 10.1016/j.colsurfb.2007.05.002 17560769

[B46] Marschner H. (1995). *Mineral Nutrition of Higher Plants.* San Diego, CA: Academic Press, 483–507. 10.1016/B978-012473542-2/50015-8

[B47] Masclaux-Daubresse C. Reisdorf-Cren M. Orsel M. (2008). Leaf nitrogen remobilisation for plant development and grain filling. *Plant Biol.* 10 23–36. 10.1111/j.1438-8677.2008.00097.x 18721309

[B48] Mobasser H. R. Mohammadi G. N. Heidari H. Abad S. Rigi K. (2014). Effect of application elements, drought stress and variety on nutrients of grain wheat in Zahak region. Iran. *J. Biodivers. Environ. Sci.* 5 105–110.

[B49] Montagu K. D. Woo K. C. (1999). Recovery of tree photosynthetic capacity from seasonal drought in the wet-dry tropics: the role of phyllode and canopy processes in *Acacia auriculiformis*. *Aust. J. Plant Physiol.* 26 135–145. 10.1071/PP98034

[B50] Nelson D. W. Sommers L. E. (1982). “Total carbon, organic carbon and organic matter,” in *Methods of Soil Analysis Chemical and Microbiological Properties* Vol. 2 ed. Page A. L. (Madison, WI: American Society of Agronomy), 539–579.

[B51] Nilsen E. T. Orcutt D. M. (1996). *Physiology of Plants Under Stress, Abiotic Factors*, 2nd Edn New York, NY: John Wiley and Sons Inc., 689.

[B52] Park J. H. Lee B. W. (2003). Photosynthetic characteristics of rice cultivars with depending on leaf senescence during grain filling. *J. Crop Sci.* 48 216–223.

[B53] Perrone I. Pagliarani C. Lovisolo C. Chitarra W. Roman F. Schubert A. (2012). Recovery from water stress affects grape leaf petiole transcriptome. *Planta* 235 1383–1396. 10.1007/s00425-011-1581-y 22241135

[B54] Roiloa S. R. Hutchings M. J. (2012). The effects of rooting frequency and position of rooted ramets on plasticity and yield in a clonal species: an experimental study with *Glechoma hederacea*. *Ecol. Res.* 27 145–152. 10.1007/s11284-011-0882-8

[B55] Sairam P. K. Deshmukh P. S. Shukla D. S. (1997). Tolerance of drought and temperature stress in relation to increased antioxidant enzyme activity in wheat. *J. Agron. Crop Sci.* 178 171–178. 10.1111/j.1439-037X.1997.tb00486.x

[B56] Saud S. Chen Y. Fahad S. Hussain S. Na L. Xin L. (2016). Silicate application increases the photosynthesis and its associated metabolic activities in Kentucky bluegrass under drought stress and post-drought recovery. *Environ. Sci. Pollut. Res.* 23 17647–17655. 10.1007/s11356-016-6957-x 27236444

[B57] Saud S. Chen Y. Longm B. Fahad S. Sadiq A. (2013). The different impact on the growth of cool season turf grass under the various conditions on salinity and draught stress. *Int. J. Agric. Sci. Res.* 3 77–84.

[B58] Saud S. Li X. Chen Y. Zhang L. Fahad S. Hussain S. (2014). Silicon application increases drought tolerance of Kentucky bluegrass by improving plant water relations and morphophysiological functions. *Sci. World J.* 2014:368694 10.1155/2014/368694 PMC4098892 25054178

[B59] Shangguan Z. P. Shao M. A. Dyckmans J. (2000). Nitrogen nutrition and water stress effects of leaf photosynthetic gas exchange and water use efficiency in winter wheat. *Environ. Exp. Bot.* 44 141–149. 10.1016/S0098-8472(00)00064-2 10996367

[B60] Shao H. B. Chu L. Y. Shao M. A. Jaleel C. A. Hongmei M. (2008). Higher plant antioxidants and redox signaling under environmental stresses. *C. R. Biol.* 331 433–441. 10.1016/j.crvi.2008.03.011 18510996

[B61] Sharp R. E. Lenoble M. E. (2002). ABA, ethylene and the control of shoot and root growth under water stress. *J. Exp. Bot.* 53 33–37. 10.1093/jxb/53.366.33 11741038

[B62] Siddique M. R. B. Hamid A. Islam M. S. (2001). Drought stress effects on water relations of wheat. *Bot. Bull. Acad. Sin.* 41 35–39.

[B63] Song C. J. Ma K. M. Qu L. Y. Liu Y. Xu X. L. Fu B. J. (2010). Interactive effects of water, nitrogen and phosphorus on the growth, biomass partitioning and water-use efficiency of *Bauhinia faberi* seedlings. *J. Arid Environ.* 74 1003–1012. 10.1016/j.jaridenv.2010.02.003

[B64] SRO (2003). *Arcadia Kentucky Bluegrass. National Turfgrass Evaluation Program, NTEP.* Tangent, OR: Seed Research of Oregon.

[B65] Tahir M. H. N. Imran M. Hussain M. K. (2002). Evaluation of sunflower (*Helianthus annuus* L.) in bred lines for drought tolerance. *Int. J. Agric. Biol.* 3 398–400.

[B66] Taiz L. Zeiger E. (2006). *Plant Physiology*, 4th Edn Sunderland, MA: Sinauer.

[B67] Taleisnik E. Peyrano G. Cordoba A. Arias C. (1999). Water retention capacity in root segments differing in the degree of exodermis development. *Ann. Bot.* 83 19–27. 10.1006/anbo.1998.0781

[B68] Teixeira E. I. George M. Herreman T. Brown H. Fletcher A. Chakwizira E. (2014). The impact of water and nitrogen limitation on maize biomass and resource-use efficiencies for radiation, water and nitrogen. *Field Crops Res.* 168 109–118. 10.1016/j.fcr.2014.08.002

[B69] UNISDR (2009). *Drought Risk Reduction Frame work and Practices: Contributing to the Implementation of the Hyogo Frame work for Action.* Geneva: United Nations Secretariat of the International Strategy for Disaster Reduction.

[B70] Vanková R. Dobrá J. Storchová H. (2012). Recovery from drought stress in tobacco: an active process associated with the reversal of senescence in some plant parts and the sacrifice of others. *Plant Signal. Behav.* 7 19–21. 10.4161/psb.7.1.18375 22301960 PMC3357359

[B71] Waraich E. A. Ahmad R. Saifullah U. Ashraf M. Y. Ehsanullah (2011). Role of mineral nutrition in alleviation of drought stress in plants. *Aust. J. Crop Sci.* 5 764–777.

[B72] Wei L. Jia L. Hu X. Zhao F. (1997). Advances in studies on the physiology and biochemistry of maize drought resistance. *Agric. Res. Arid Areas* 15 66–71.

[B73] Yang S. Vanderbeld B. Wan J. Huang Y. (2010). Narrowing down the targets: towards successful genetic engineering of drought-tolerant crops. *Mol. Plant* 3 469–490. 10.1093/mp/ssq016 20507936

[B74] Zhang L. X. Li S. X. Zhang H. Liang Z. S. (2007). Nitrogen rates and drought stress effects on production, lipid peroxidation and antioxidative enzyme activities in two maize (*Zea mays* L.) genotypes. *J. Agron. Crop Sci.* 193 387–397. 10.1111/j.1439-037X.2007.00276.x

